# Fin56-induced ferroptosis is supported by autophagy-mediated GPX4 degradation and functions synergistically with mTOR inhibition to kill bladder cancer cells

**DOI:** 10.1038/s41419-021-04306-2

**Published:** 2021-10-29

**Authors:** Yadong Sun, Niklas Berleth, Wenxian Wu, David Schlütermann, Jana Deitersen, Fabian Stuhldreier, Lena Berning, Annabelle Friedrich, Seda Akgün, María José Mendiburo, Sebastian Wesselborg, Marcus Conrad, Carsten Berndt, Björn Stork

**Affiliations:** 1 Institute of Molecular Medicine I, Medical Faculty, Heinrich Heine University, Düsseldorf, Germany; 2 Institute of Metabolism and Cell Death, Helmholtz Zentrum München, Neuherberg, Germany; 3 Pirogov Russian National Research Medical University, Laboratory of Experimental Oncology, Ostrovityanova 1, Moscow, 117997 Russia; 4 Department of Neurology, Medical Faculty, Heinrich Heine University, Düsseldorf, Germany

**Keywords:** Macroautophagy, Macroautophagy

## Abstract

Ferroptosis is a form of regulated cell death that emerges to be relevant for therapy-resistant and dedifferentiating cancers. Although several lines of evidence suggest that ferroptosis is a type of autophagy-dependent cell death, the underlying molecular mechanisms remain unclear. Fin56, a type 3 ferroptosis inducer, triggers ferroptosis by promoting glutathione peroxidase 4 (GPX4) protein degradation via a not fully understood pathway. Here, we determined that Fin56 induces ferroptosis and autophagy in bladder cancer cells and that Fin56-triggered ferroptosis mechanistically depends on the autophagic machinery. Furthermore, we found that autophagy inhibition at different stages attenuates Fin56-induced oxidative stress and GPX4 degradation. Moreover, we investigated the effects of Fin56 in combination with Torin 2, a potent mTOR inhibitor used to activate autophagy, on cell viability. We found that Fin56 synergizes with Torin 2 in cytotoxicity against bladder cancer cells. Collectively, our findings not only support the concept that ferroptosis is a type of autophagy-dependent cell death but imply that the combined application of ferroptosis inducers and mTOR inhibitors is a promising approach to improve therapeutic options in the treatment of bladder cancer.

## Introduction

Bladder cancer (BC) is the 10th most-common cancer worldwide, with an estimated 549,000 new cases and 200,000 deaths each year [1]. Recently, tumor genomic profiling supports the identification of promising therapeutic targets [2, 3]. Among them, the phosphatidylinositol 3-kinase (PI3K)/AKT/mammalian target of rapamycin (mTOR) pathway regulates various processes, including proliferation, survival, and autophagy [4, 5]. In up to 40% of BCs, mTOR signaling is activated and related to tumor progression [6, 7]. Whereas many mTOR inhibitors are in different phases of (pre-)clinical trials [8], the monotherapy of these inhibitors has not yet led to effective, desirable outcomes.

Ferroptosis, an iron-dependent form of regulated cell death, is entirely distinct from other cell death modalities [9]. Although ferroptosis has been linked to various pathological scenarios, it has received extensive attention in cancer therapy research [10]. Since cancer cells require higher levels of iron, generate higher levels of reactive oxygen species (ROS), and have an altered lipid metabolism compared with normal cells, they become more susceptible to ferroptosis [11]. So far, three types of inducers are available, which employ different modes of action [12]. These compounds have in common that they inhibit glutathione peroxidase 4 (GPX4), which itself represents a key inhibitor of phospholipid peroxidation [13]. GPX4 inhibition can be achieved either indirectly via depletion of glutathione by inhibiting cysteine uptake (e.g., type 1 inducer erastin) or directly (e.g., type 2 inducer RAS-selective lethal 3, RSL3). Fin56, a recently developed type 3 inducer, triggers ferroptosis by promoting degradation of GPX4 via a not fully understood pathway [14]. Accumulating evidence suggests that ferroptosis is related to another important cellular process, i.e., autophagy [15, 16].

Autophagy has an important role in transporting unfolded proteins and damaged organelles into lysosomes to be recycled [17]. Autophagy can be subdivided into three morphologically distinct types: macroautophagy, microautophagy, and chaperone-mediated autophagy (CMA) [18]. The machinery regulating macroautophagy (hereafter, referred to as autophagy) includes the unc-51-like kinase 1 (ULK1) complex, the class III PI3K lipid kinase complex, and two ubiquitin-like conjugation systems [19]. mTOR is one of the main upstream regulators of autophagy. It phosphorylates ULK1 at Ser758 (Ser757 in murine ULK1) and thereby suppresses autophagy [20]. Once mTOR is pharmacologically inhibited or cells are kept under starvation conditions, this ULK1 site becomes rapidly dephosphorylated, leading to ULK1 activation and the induction of autophagy [21]. Although autophagy is generally considered a pro-survival mechanism, its role in cancer biology is more complex [22, 23]. In brief, autophagy is considered to execute a protective function during tumorigenesis; once tumor cells are established, autophagy supports tumor survival and metastasis. Moreover, with the development of methods to monitor autophagic flux in various contexts, autophagy-dependent cell death is considered as a genuine death pathway, making the relationship between autophagy and cancer biology even more complex [24, 25].

Recent evidence suggests that autophagy facilitates ferroptosis via the selective removal of ferroptosis-regulating molecules [26–28]. These pathways include NCOA4-mediated ferritinophagy, RAB7A-mediated lipophagy, or p62/SQSTM1-mediated clockophagy [26, 28]. Furthermore, it has been observed that erastin and RSL3 induce autophagic flux and also affect GPX4 levels [26, 28]. It has been reported that erastin promotes the degradation of GPX4 via CMA [29], and that RSL3 can block mTOR activation and cause GPX4 degradation in pancreatic cancer cells [30]. Along these lines, pharmacologic inhibition of mTORC1 decreases GPX4 protein levels [31], further supporting the relationship between autophagy and ferroptosis.

It has been described that Fin56 promotes the reduction of GPX4 but the mechanism or a possible link to autophagy remain unclear [14, 32]. Here, we found that Fin56 initiates ferroptosis and autophagy in BC cells. We observed that Fin56-induced autophagy promotes the degradation of GPX4 and ferritin, whereas the inhibition of autophagy dampened Fin56-induced lipid peroxidation and ferroptosis. In addition, we provide evidence that the combination of Fin56 with the mTOR inhibitor Torin 2 has a synergistic effect in effectively killing BC cells. In sum, we propose that the combined use of ferroptosis inducers and mTOR inhibitors is a promising approach to improve therapeutic options in the treatment of BC.

## Results

### Fin56 induces autophagy-associated cell death in BC cells

**Fig. 1:**
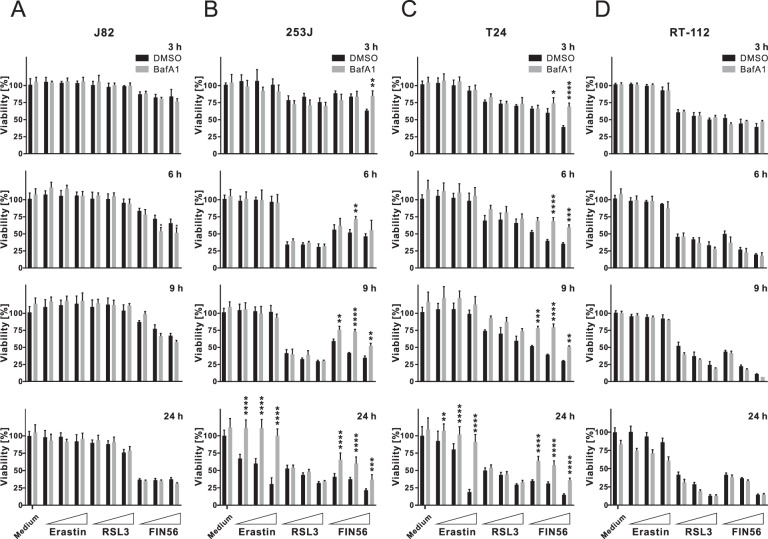
Fin56 induces autophagy-associated cell death in bladder cancer cells. **A**–**D** J82, 253J, T24, and RT-112 bladder cancer cells were treated with different concentrations (1 µM, 2 µM, and 5 µM) of Erastin, RSL3, and Fin56 with or without 20 nM bafilomycin A_1_ (BafA1) for different times (3, 6, 9, and 24 h). After treatment, cell viability was measured using MTT assay (**A**–**D** represent the cell viability of J82, 253J, T24, and RT-112, respectively). Results are shown as means + SD of two independent experiments. *P* values were determined by two-way ANOVA with Sidak’s post hoc test. *p < 0.05; **p < 0.01; ***p < 0.001; ****p < 0.0001 (comparison between DMSO- and BafA1-treated samples).

It has been reported that the susceptibility to ferroptosis varies in different cancer cells [33, 34]. To evaluate the effects of ferroptosis inducers on human BC cells, we employed type 1, 2, and 3 inducers (erastin, RSL3 and Fin56) and four BC cell lines representing different clinical stages and pathological grades of BC, i.e., J82, 253J, T24 and RT-112 cells (Figure S1A). All cell lines express GPX4 and solute carrier family 7 member 11 (SLC7A11) (Figure S1B). The cystine/glutamate antiporter system x_c_^−^ plays a key role in importing cystine for glutathione synthesis and thus protects from oxidative stress and ferroptosis [35]. The expression level of its subunit SLC7A11 directly correlates with the activity of the antiporter [36]. Owing to its mode of action, erastin failed to induce cell death in all tested cell lines after 3–9 h of treatment, whereas RSL3 and Fin56 triggered cell death in BC cells except for J82 (Fig. 1). After 24 h, erastin induced cell death in 253J and T24 cells, and Fin56 induced cell death in all tested cell lines. Moreover, cell death caused by Fin56 was dampened by the autophagy inhibitor bafilomycin A_1_ in 253J and T24 cells, indicating that Fin56 might trigger autophagy-associated cell death in these BC cells (Fig. 1B, C).

### Fin56 induces ferroptosis in BC cells

**Fig. 2:**
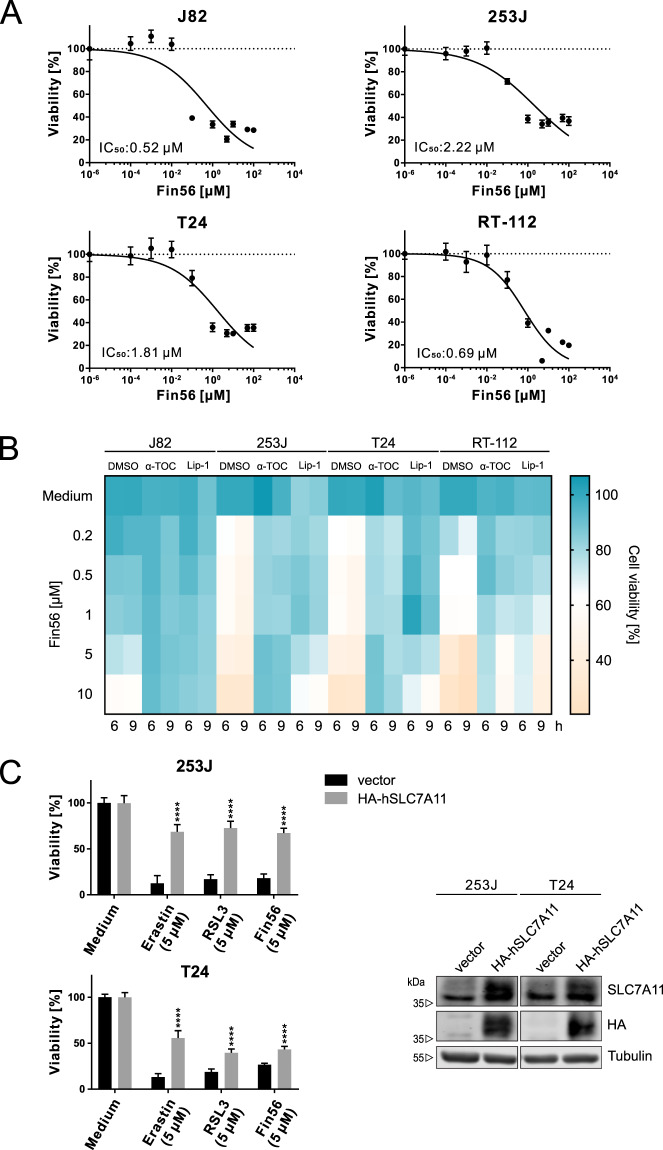
Fin56 induces ferroptosis in bladder cancer cells. **A** J82, 253J, T24, and RT-112 bladder cancer cells were treated with different concentrations (0.1 nM–100 µM) of Fin56 for 72 h. After treatment, cell viability was measured using MTT assay. Results are shown as means ± SD of two independent experiments performed in quadruplicate for each treatment. **B** Cell viability heatmap of the effects of different ferroptosis inhibitors on cell death induced by Fin56. J82, 253J, T24, and RT-112 were treated with indicated concentrations of Fin56 with or without α-tocopherol (α-TOC, 100 µM) or liproxstatin-1 (Lip-1, 500 nM) for 6 or 9 h. After treatment, cell viability was measured using MTT assay. Results are from two independent experiments performed in triplicates for each treatment. The corresponding histograms and statistical analyses are shown in supplementary figure S1. **C** HA-hSLC7A11-overexpressing 253J or T24 cells and their corresponding vector control cells were treated with 5 µM erastin, RSL3, or Fin56 for 24 h. After treatment, cell viability was measured using MTT assay. Results are shown as means + SD of two independent experiments performed in triplicates for each treatment. *P* values were determined by two-way ANOVA with Sidak’s post hoc test. ****p < 0.0001 (comparison between vector- and *HA-hSLC7A11*-transfected cells). In order to confirm the overexpression of HA-hSLC7A11, cells were lysed and cellular lysates were immunoblotted for SLC7A11, HA, and tubulin.

To further characterize Fin56-induced cell death, we treated BC cell lines with increasing concentrations of Fin56 for 72 h. Fin56 conferred high cytotoxicity in all tested cell lines (Fig. 2A). In order to further investigate the mode of cell death, we next treated the four BC cell lines with Fin56 in the presence or absence of two different ferroptosis inhibitors (α-tocopherol and liproxstatin-1). Both inhibitors significantly prevented Fin56-induced cell death in all BC cell lines (Fig. 2B and S2).

Since Fin56 induces autophagy-associated cell death in 253J and T24 cells, we focused on these two cell lines. We established SLC7A11-overexpressing 253J and T24 cells and exposed them to different ferroptosis inducers. Compared to control cells, SLC7A11 overexpression inhibited cell death induced by the three inducers (Fig. 2C). Taken together, these results suggest that Fin56 induces ferroptosis in these BC cells.

### Fin56 induces autophagy in BC cells

**Fig. 3:**
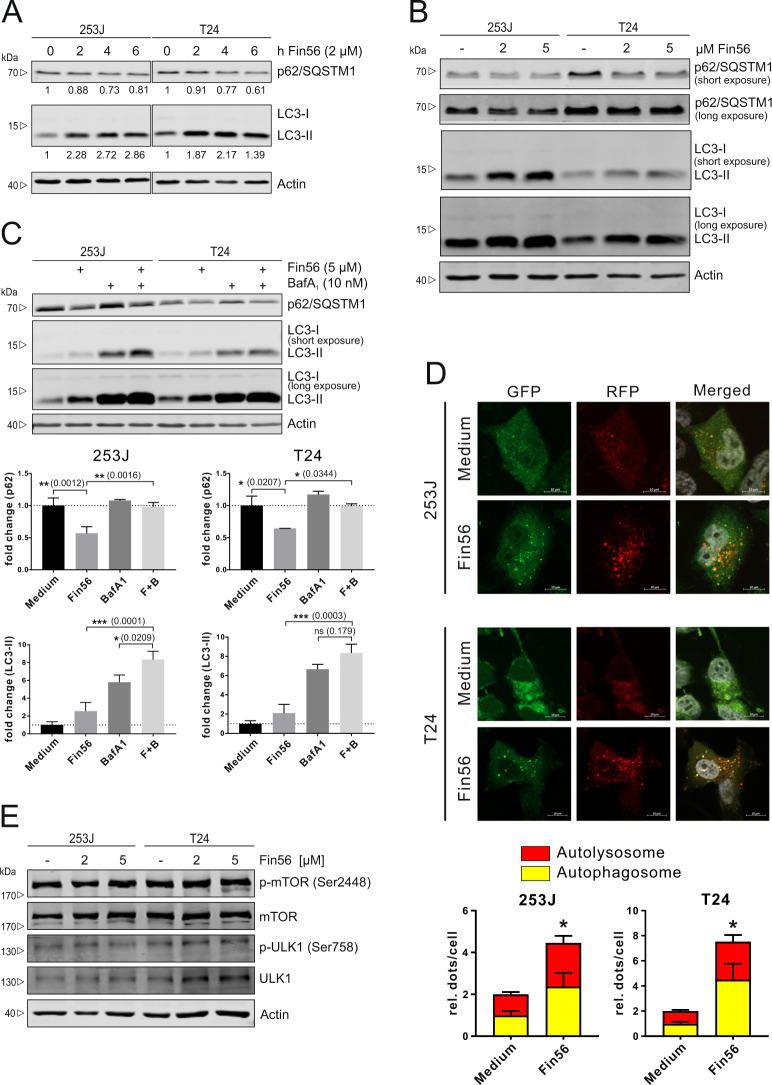
Fin56 induces autophagy in bladder cancer cells. **A** 253J and T24 cells were treated with 2 µM Fin56 for 0, 2, 4, or 6 h. After incubation, cells were lysed and cellular lysates were immunoblotted for the indicated proteins. One representative immunoblot is shown. The quantifications are from two independent experiments. **B** 253J and T24 cells were treated with 2 or 5 µM Fin56 for 6 h. After incubation, cells were lysed and cellular lysates were immunoblotted for the indicated proteins. One representative immunoblot of three independent experiments is shown. **C** 253J and T24 cells were treated with Fin56 (5 µM), BafA1 (10 nM), or Fin56 + BafA1 (F + B) for 6 h. After the incubation, cells were lysed and cellular lysates were immunoblotted for the indicated proteins. One representative immunoblot is shown. The quantifications of indicated ratios are from three independent experiments (means + SD). The respective *P* values are depicted in the diagram. **D** 253J and T24 stably expressing mRFP-EGFP-rLC3 cells were grown on glass coverslips one day prior to treatment. The following day, cells were treated with 2 µM Fin56 for 4 h and fixed after treatment. Red puncta represent autolysosomes and yellow puncta represent autophagosomes. One representative image is shown. Scale bars are 10 µm. The quantifications of puncta are from two independent experiments (means + SD); a minimum of 112 cells per stimulation was analyzed. The respective *P* values are depicted in the diagram (comparison of mRFP^+^ EGFP^+^ puncta between medium- and Fin56-treated cells). **E** 253J and T24 cells were treated with 2 µM or 5 µM Fin56 for 6 h. After the incubation, cells were lysed and cellular lysates were immunoblotted for the indicated proteins. One representative immunoblot of three independent experiments is shown.

Since bafilomycin A_1_ partially protected cells from Fin56-induced ferroptosis, we investigated whether Fin56 treatment induced autophagy. Autophagy was evaluated by determining the changes of the autophagosome marker LC3 and the autophagy receptor sequestosome 1 (SQSTM1/p62) by immunoblotting [37]. Levels of LC3-II, a membrane-associated form of LC3, were increased after Fin56 treatment in a time- and dose-dependent manner. Simultaneously, SQSTM1/p62 was decreased in the same manner (Fig. 3A, B). These results indicate that Fin56 treatment can induce autophagy in BC. However, the accumulation of LC3-II may represent either an increase in autophagosome generation and/or a block of autophagic flux. To further analyze this, we performed LC3 and SQSTM1/p62 turnover assays using bafilomycin A_1_ to block lysosomal degradation of autophagosomes. We found that LC3-II levels increased under Fin56 treatment or bafilomycin A_1_ treatment alone. However, the combined treatment with Fin56 and bafilomycin A_1_ led to an increased accumulation of LC3-II (Fig. 3C). Similar results were obtained from the SQSTM1/p62 turnover assay. These results suggest that Fin56 increases autophagic flux. However, since LC3 and SQSTM1/p62 can be transcriptionally regulated, autophagic flux should not only be assessed by their protein abundance [38]. Thus, we generated 235J and T24 cells stably expressing mRFP-EGFP-rLC3, and monitored autophagic flux by the detection of yellow dots (mRFP/EGFP colocalization, representing autophagosomes) and red dots (mRFP only, representing autolysosomes) using fluorescence microscopy [39]. Consistently, Fin56 treatment increased the number of both autophagosomes and autolysosomes, indicating increased autophagic flux in 253J and T24 cells (Fig. 3D).

Since mTOR is a central upstream regulator of autophagy [40], we next investigated whether mTOR was involved in Fin56-induced autophagy. We analyzed mTOR phosphorylation at Ser2448 and ULK1 phosphorylation at Ser758, which both have been linked to autophagy inhibition [41, 42]. Fin56 treatment, however, did not affect phosphorylation levels of mTOR and ULK1 at these sites (Fig. 3E), indicating that Fin56 induces autophagy in BC through an mTOR-independent pathway.

### Fin56 induces autophagy-dependent ferroptosis

**Fig. 4:**
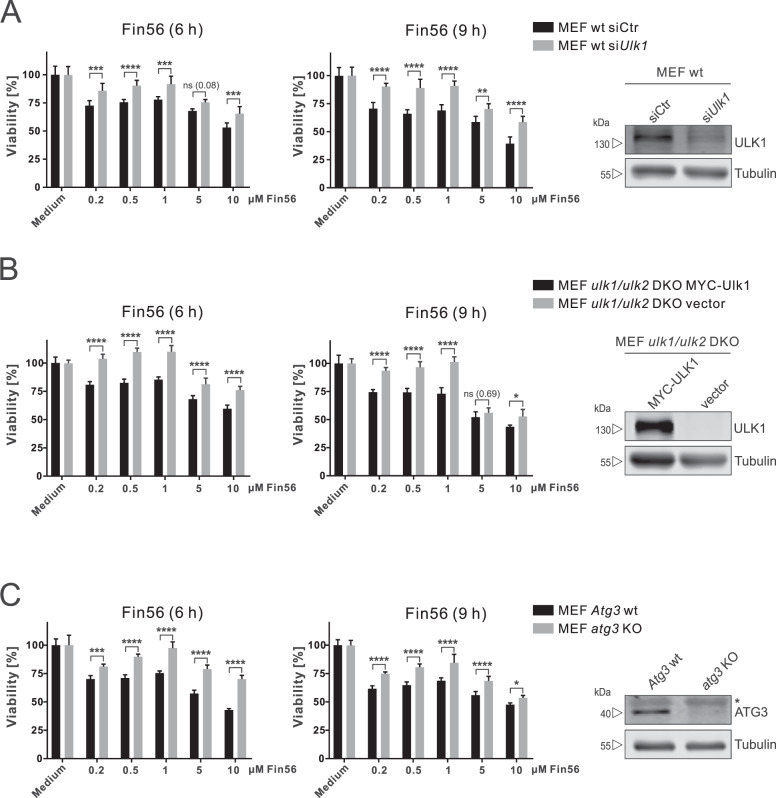
Autophagy inhibition attenuates Fin56-induced ferroptosis. **A** MEFs were transfected with control or *Ulk1* siRNAs. After 48 h, cells were treated with different concentrations of Fin56 (0.2 µM–10 µM) for 6 or 9 h. After treatment, cell viability was measured using MTT assay. Results are shown as means + SD of two independent experiments performed in triplicates for each treatment. *P* values were determined by two-way ANOVA with Sidak’s post hoc test. **B** *ulk1/ulk2* DKO MEFs and *ulk1/ulk2* DKO MEFs reconstituted with MYC-ULK1 were treated with different concentrations of Fin56 (0.2 µM–10 µM) for 6 and 9 h. After treatment, cell viability was measured using MTT assay. Results are shown as means + SD of two independent experiments performed in triplicates for each treatment. *P* values were determined by two-way ANOVA with Sidak’s post hoc test. **C** *atg3* KO and *Atg3* WT MEFs were treated with different concentrations of Fin56 (0.2 µM–10 µM) for 6 and 9 h. After treatment, cell viability was measured using MTT assay. Results are shown as means + SD of two independent experiments performed in triplicates for each treatment. *P* values were determined by two-way ANOVA with Sidak’s post hoc test. *p < 0.05; **p < 0.01; ***p < 0.001; ****p < 0.0001. In order to confirm knockdown or knockout, cells were lysed and cellular lysates were immunoblotted for ULK1, ATG3, and tubulin. The asterisk in **C** indicates an unspecific background band.

To investigate the relationship between Fin56-induced autophagy and ferroptosis more comprehensively, we inhibited autophagy by using knockdown or/and knockout of ULK1 and ATG3 and investigated Fin56-induced ferroptosis. ULK1 depletion blocks autophagy initiation, while in the absence of ATG3 autophagy is impaired during autophagosome biogenesis. We found that siRNA-mediated knockdown of ULK1 decreased Fin56-induced ferroptosis compared with control siRNA (Fig. 4A). We also employed *ulk1/ulk2* double-knockout (DKO) murine embryonic fibroblasts (MEFs) and *ulk1/ulk2* DKO MEFs reconstituted with MYC-ULK1 to confirm the siRNA results. Compared with reconstituted cells, *ulk1/ulk2* DKO cells had higher cell viability after treatment with different concentrations of Fin56 (Fig. 4B). Moreover, autophagy deficiency has been confirmed in cells lacking ATG3, an E2-like enzyme mediating LC3 lipidation [43]. In line with the ULK1 results, *atg3* KO cells were more viable than *Atg3* WT cells upon Fin56 treatment (Fig. 4C). Taken together, these results suggest that Fin56 triggers autophagy-dependent ferroptosis.

### Autophagy inhibition attenuates Fin56-induced oxidative stress and lipid peroxidation

**Fig. 5:**
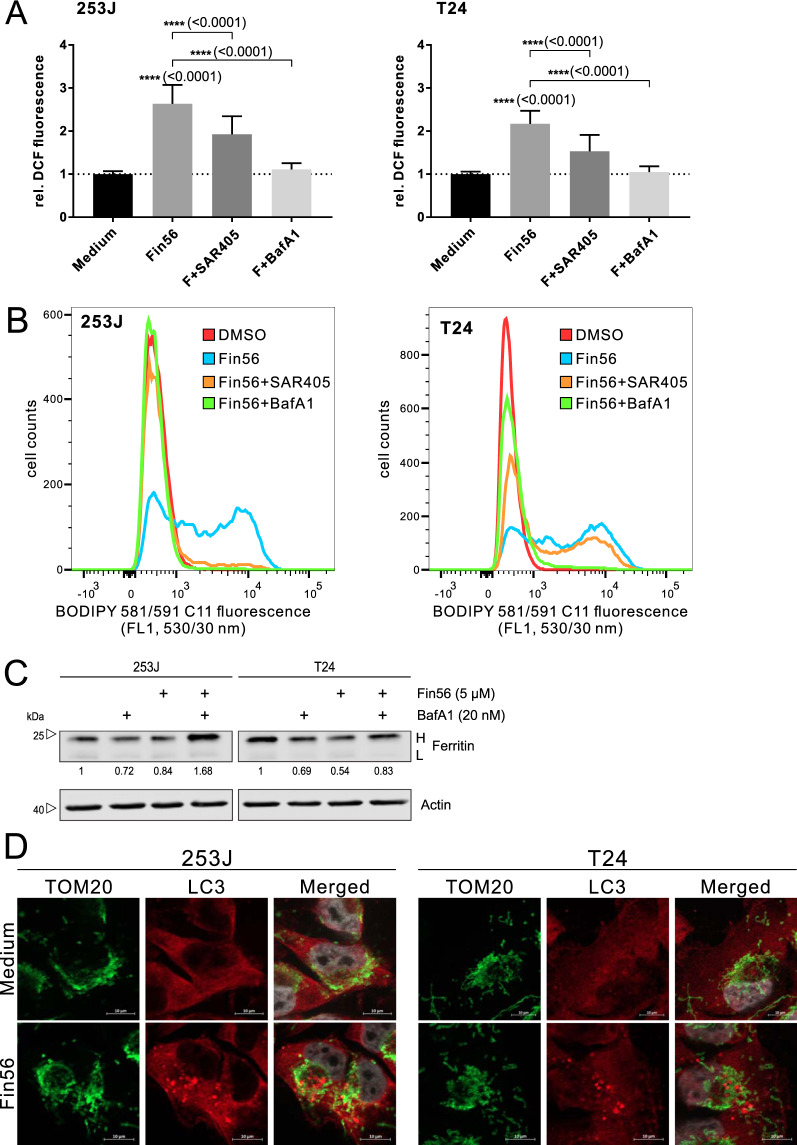
Autophagy inhibition attenuates Fin56-induced oxidative stress. **A** 253J and T24 cells were treated with Fin56 (2 µM), Fin56 + SAR405 (2 µM) or Fin56 + BafA1 (20 nM) for 4 h. After treatment, cells were treated with 10 μM H_2_DCFDA for 30 min. After that, DCF fluorescence was measured. Results are shown as means + SD of three independent experiments performed in triplicates for each treatment. *P* values were determined by one-way ANOVA with Tukey´s post hoc test. The respective *P* values are depicted in the diagram. **B** 253J and T24 cells were treated with DMSO, Fin56 (2 µM), Fin56 + SAR405 (2 µM) or Fin56 + BafA1 (20 nM) for 4 h. Then, cells were incubated with 1.5 µM BODIPY 581/591 C11 for 30 min at 37 °C before they were harvested by trypsinisation. Subsequently, cells were resuspended in 500 μL PBS and analyzed by flow cytometry. Data were collected using the FL1 detector with a 530/30 BP filter. A total of 20,000 events was acquired for each sample. **C** 253J and T24 cells were treated with Fin56 (5 µM), BafA1 (20 nM), or Fin56 + BafA1 for 4 h. After the incubation, cells were lysed and cellular lysates were immunoblotted for the indicated proteins (H, heavy chain; L, light chain). One representative immunoblot is shown. The quantifications are from two independent experiments. **D** 253J and T24 cells were grown on glass coverslips one day prior to treatment. The following day, cells were treated with 5 µM Fin56 for 6 h. After treatment, cells were fixed, permeabilized, and incubated with indicated antibodies. Nuclei were stained with DAPI. One representative image is shown.

Lipid peroxidation is one of the hallmarks of ferroptosis. Frequently, the 2’,7’-dichlorofluorescein (DCF) assay is used to evaluate cellular oxidative stress. As noted by Karlsson et al. [44], the oxidation of H_2_DCF to DCF requires the presence of both H_2_O_2_ and redox-active transition metals such as iron, and rather indicates the impact of hydroxyl radicals generated during Fenton-type reactions. Since non-enzymatic lipid peroxidation is driven by the Fenton reaction, we performed the DCF assay and observed that Fin56 treatment increased DCF-induced fluorescence (Fig. 5A). Moreover, autophagy inhibition at different stages by using either bafilomycin A_1_ or the class III PI3K inhibitor SAR405 reduced Fin56-induced DCF fluorescence. We also monitored lipid peroxidation by flow cytometric analysis of BODIPY 581/591 C11 fluorescence. In both 253J and T24 cells, SAR405 and bafilomycin A_1_ reversed the Fin56-induced increase in the 530/30 nm fluorescence (Fig. 5B), indicating that autophagy inhibition attenuates Fin56-induced lipid peroxidation. We also analyzed BODIPY 581/591 C11-stained cells by immunofluorescence. Notably, we observed “onion-like” structures in Fin56-treated cells that were not present in control-treated cells (Figure S3). In order to investigate the potential contribution of intracellular iron, we next analyzed the expression of ferritin, an intracellular iron storage protein that sequesters iron in a soluble/non-toxic form [45]. We found that Fin56 promoted ferritin degradation and that autophagy inhibition interfered with its degradation (Fig. 5C). Excess oxidative stress causes mitochondrial damage and triggers the selective clearance of damaged mitochondria by mitophagy [46]. However, Fin56 did not induce significant mitochondrial fragmentation or mitophagy, as observed by staining of TOM20 and LC3 by immunofluorescence (Fig. 5D). Collectively, Fin56 induces oxidative damage/lipid peroxidation that can be prevented by the inhibition of autophagy and does not affect mitochondrial integrity.

### Autophagy inhibition attenuates Fin56-induced GPX4 degradation

**Fig. 6:**
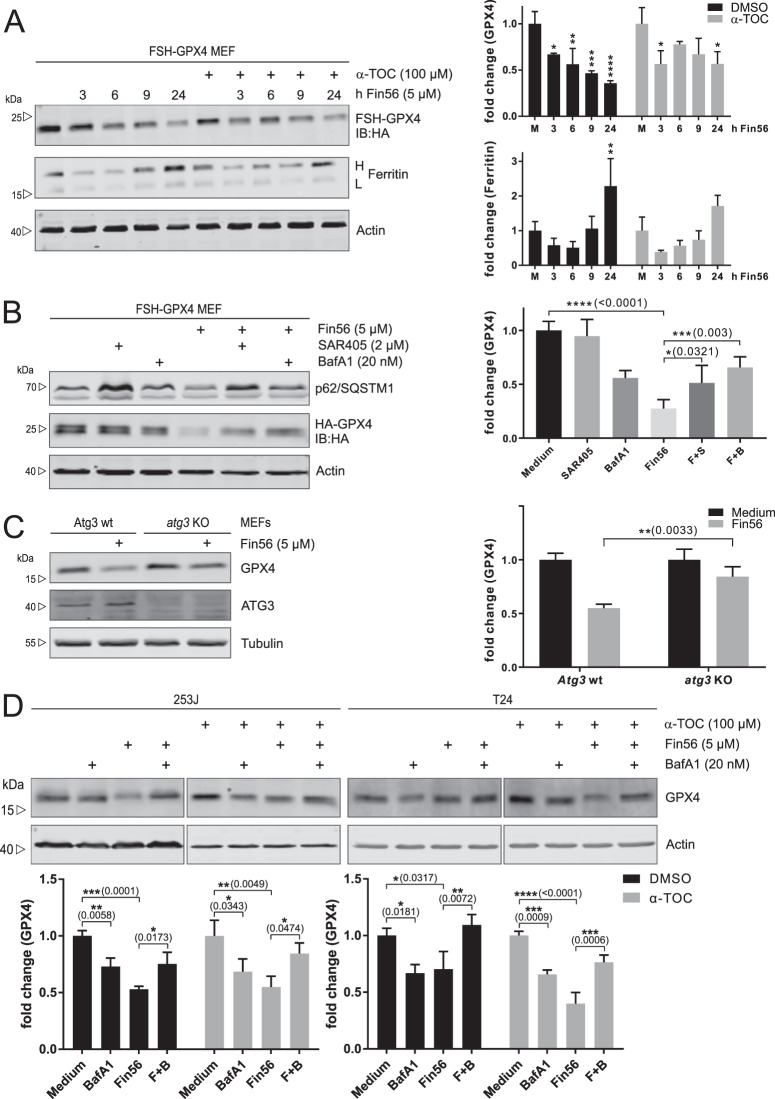
Autophagy inhibition attenuates Fin56-induced GPX4 degradation. **A** FSH-GPX4 MEFs were left untreated (medium, M) or were treated with 5 µM Fin56 with or without 100 µM α-tocopherol (α-TOC) for 3, 6, 9, or 24 h. After the incubation, cells were lysed and cellular lysates were immunoblotted for the indicated proteins. One representative immunoblot is shown. The quantifications are from three independent experiments. *P* values were determined by two-way ANOVA with Tukey´s post hoc test (comparison always to medium-treated control). H: heavy chain; L: light chain. **B** FSH-GPX4 MEFs were treated with Fin56 (5 µM), SAR405 (2 µM), BafA1 (20 nM), Fin56 + SAR405 (F + S) or Fin56 + BafA1 (F + B) for 24 h. After the incubation, cells were lysed and cellular lysates were immunoblotted for the indicated proteins. One representative immunoblot is shown. The quantifications are from five independent experiments. *P* values were determined by one-way ANOVA with Tukey´s post hoc test. **C** *Atg3* wild-type or *atg3* KO MEFs were treated with 5 µM Fin56 for 24 h. After the incubation, cells were lysed and cellular lysates were immunoblotted for the indicated proteins. One representative immunoblot is shown. The quantifications are from three independent experiments. *P* values were determined by two-way ANOVA with Sidak’s post hoc test. **D** 253J and T24 cells were treated with Fin56 (5 µM), BafA1 (20 nM) or Fin56 + BafA1 (F + B) with or without 100 µM of α-TOC for 24 h. After the incubation, cells were lysed and cellular lysates were immunoblotted for the indicated proteins. The quantifications are from three independent experiments. *P* values were determined by one-way ANOVA with Tukey’s post hoc test. *p < 0.05; **p < 0.01; ***p < 0.001; ****p < 0.0001.

It has been reported that one pathway of Fin56-triggered ferroptosis is executed via GPX4 degradation [14], but the mechanistic details remained to be understood. We employed FLAG-Strep-HA-GPX4 (FSH-GPX4)-expressing MEFs to further investigate the Fin56-mediated GPX4 regulation [47, 48]. In addition, we used the antioxidant α-tocopherol to exclude the possibility that changes in GPX4 abundance are downstream consequences of the increased formation of the hydroxyl radical. We observed that Fin56 decreased the abundance of both ferritin and GPX4 in a time-dependent manner which was insensitive to α-TOC (Fig. 6A). Interestingly, we also found that ferritin levels increased after 9 h of Fin56 treatment. We speculate that the late increase in ferritin abundance may represent a self-protective mechanism that promotes the conversion of cellular iron into a non-toxic form to alleviate prolonged oxidative damage. Next, we investigated if autophagy is involved in this Fin56-induced GPX4 degradation, and indeed, we found that autophagy inhibition by bafilomycin A_1_ or SAR405 attenuated GPX4 degradation induced by Fin56 in FSH-GPX4 MEFs (Fig. 6B). As it has been reported that erastin can induce GPX4 degradation via CMA [29], we next wanted to know if indeed macroautophagy is the mechanism mediating Fin56-induced GPX4 degradation. In a first approach, we again used the autophagy-incompetent *atg3* KO MEFs. We observed that Fin56-induced GPX4 degradation is largely blocked in *atg3* KO MEFs (Fig. 6C). In an alternative approach, we investigated the colocalization of GPX4 and LC3 by immunofluorescence. We found that treatment of FSH-GPX4-expressing MEFs with Fin56 induces the colocalization of GPX4 and LC3 (Figure S4). As both ATG3 and LC3 are not required for CMA, we think that Fin56-induced GPX4 degradation depends on macroautophagy. Finally, we investigated if Fin56 also induces GPX4 degradation in the BC cell lines 253J and T24, and—if yes—if this can be blocked by autophagy inhibition. We observed that both were the case (Fig. 6D). Taken together, Fin56 induces GPX4 degradation in MEFs and BC cells, and inhibition of autophagy can block this degradation.

**Fig. 7:**
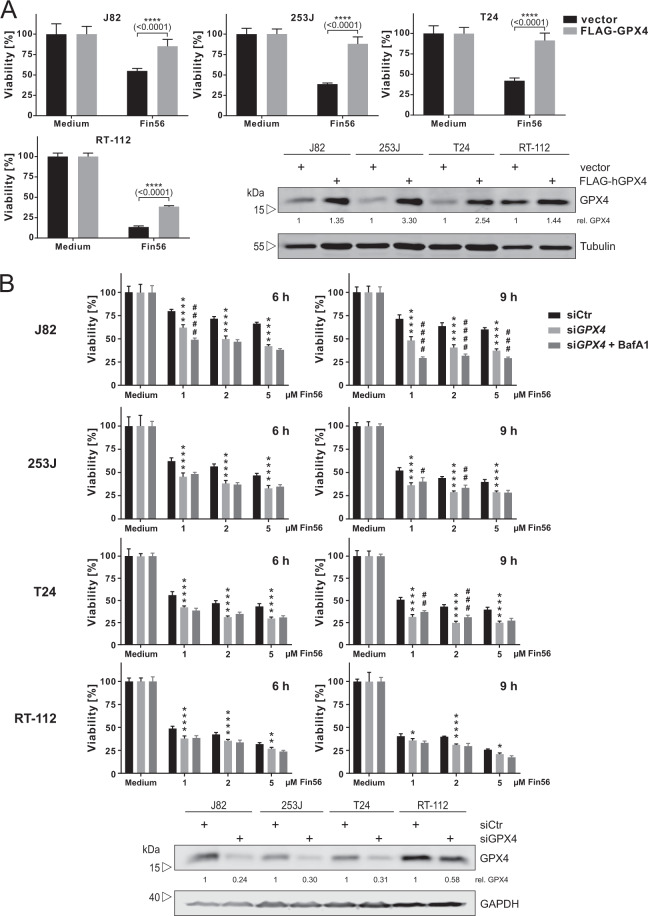
GPX4 knockdown abolishes autophagy-dependency of Fin56-induced ferroptosis. **A** J82, 253J, T24, or RT-112 cells were transiently transfected with empty vector or cDNA encoding FLAG-hGPX4. After 30 h, cells were seeded into 96-well plates for MTT cell viability assay and into six-well plates for immunoblotting. For cell viability assay, cells were treated with 5 µM Fin56 for 9 h. Then, cell viability was measured using MTT assay. Results are shown as means + SD of two independent experiments performed in triplicates for each treatment. *P* values were determined by two-way ANOVA with Sidak’s post hoc test. Simultaneously, cells in six-well plates were lysed and cellular lysates were immunoblotted for GPX4 and tubulin. **B** J82, 253J, T24, or RT-112 cells were transfected with control or *GPX4* siRNAs. After 48 h, cells were left untreated or treated with different concentrations of Fin56 (1–5 µM) with or without 20 nM bafilomycin A_1_ for 6 or 9 h. After treatment, cell viability was measured using MTT assay. Results are shown as means + SD of two independent experiments performed in triplicates for each treatment. *P* values were determined by one-way ANOVA with Tukey’s post hoc test. The asterisk (*) represents the comparison between siCtr and si*GPX4*, whereas the octothorpe (#) indicates the comparison between si*GPX4* and si*GPX4* + BafA1. Successful knockdown was confirmed by immunoblotting for GPX4 and GAPDH.

As we found that autophagy inhibition partially reduces Fin56-induced cell death (Fig. 1) and GPX4 degradation (Fig. 6), we next wanted to know if these observations are correlated. First, we overexpressed human GPX4 in all four BC cell lines, and we detected a reduction of Fin56-induced cell death (Fig. 7A), confirming that the regulation of GPX4 levels is central to the control of ferroptosis in these cell lines. In RT-112 cells, which express high GPX4 levels endogenously (Fig. 7A and S1B), Fin56-induced cell death was not completely preventable by GPX4 overexpression, supporting a previous report describing an additional Fin56-induced ferroptosis pathway independent of GPX4 degradation [14]. Next, we silenced GPX4 in all four BC cell lines (Fig. 7B). Notably, Fin56-induced reduction of cell viability was not sensitive to bafilomycin A_1_ in 253J and T24 cells anymore, and silencing did not sensitize J82 and RT-112 cells. Apparently, the siRNA-mediated knockdown of GPX4 (transcriptional control) abolishes the autophagy-dependent part of Fin56-induced ferroptosis (control on the protein level). These data indicate that the sensitivity of 253J and T224 cells to bafilomycin A_1_ is—at least partially—caused by the Fin56-induced autophagic degradation of GPX4.

### mTOR inhibition synergistically sensitizes BCs to Fin56-induced ferroptosis

**Fig. 8:**
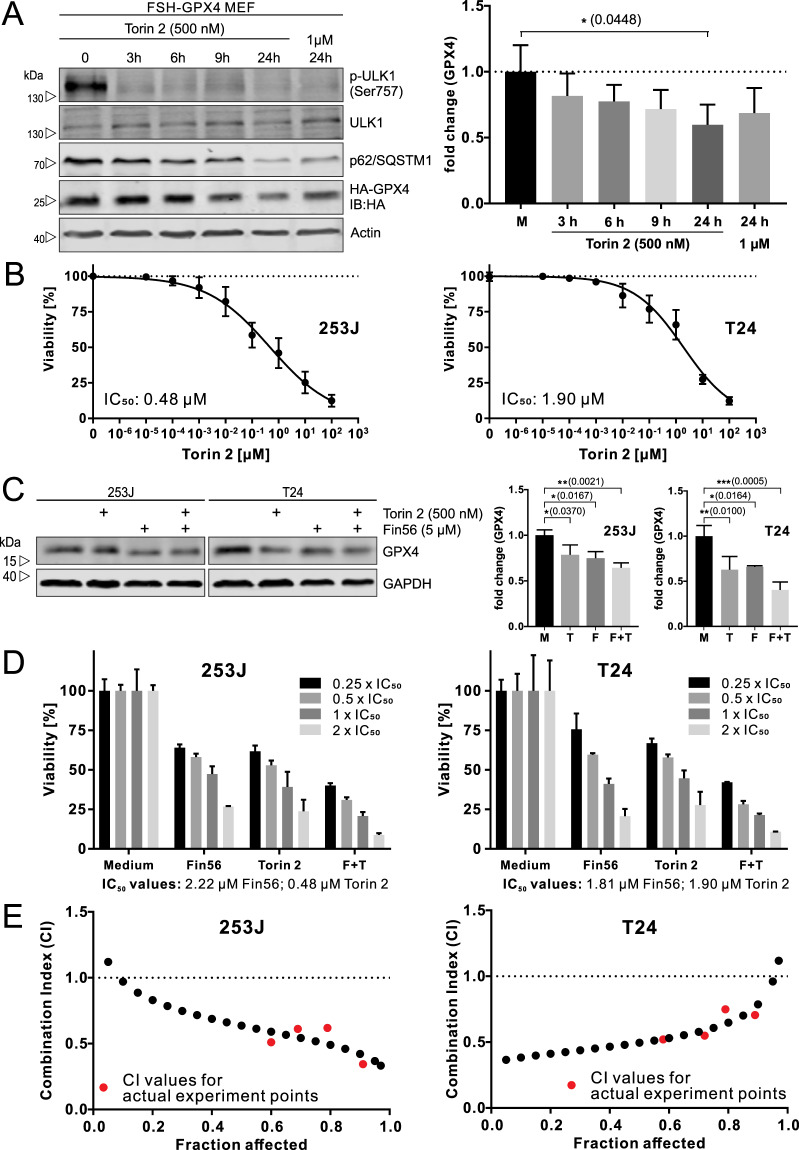
mTOR inhibition synergistically sensitizes BCs to Fin56-induced ferroptosis. **A** FSH-GPX4 MEFs were left untreated (medium, M) or were treated with 500 nM Torin 2 for 3, 6, 9, or 24 h, or with 1 µM Torin 2 for 24 h. After the incubation, cells were lysed and cellular lysates were immunoblotted for the indicated proteins. One representative immunoblot is shown. The quantifications are from three independent experiments. *P* values were determined by one-way ANOVA with Dunnett´s post hoc test. **B** 253J and T24 cells were treated with different concentrations (0.1 nM–100 µM) of Torin 2 for 72 h. After treatment, cell viability was measured using MTT assay. Results are shown as means ± SD of three independent experiments performed in triplicates for each treatment. **C** 253J and T24 cells were left untreated (medium, M) or were treated with the indicated treatment (500 nM Torin 2, T; 5 µM Fin56, F) for 9 h. After the incubation, cells were lysed and cellular lysates were immunoblotted for the indicated proteins. The quantifications are from three independent experiments. *P* values were determined by one-way ANOVA with Tukey’s post hoc test. **D** 253J and T24 cells were treated with 0.25×, 0.5×, 1×, or 2× of the IC_50_ values of the single substances for 72 h. After treatment, cell viability was measured using MTT assay. Results are shown as means + SD of two independent experiments performed in sextuplicates for each treatment. **E** Combination indices (CI) were calculated using the software CompuSyn. Synergism (CI < 1), additivism (CI = 1) and antagonism (CI > 1).

As autophagy promotes Fin56-induced ferroptosis, we hypothesized that triggering both processes simultaneously might be a valuable strategy to increase the efficacy of killing BC cells. Here, we used mTOR inhibition to induce autophagy. We first validated that the mTOR inhibitor Torin 2 worked appropriately in our cellular systems. In FSH-GPX4-expressing MEFs, Torin 2 inhibited ULK1 Ser757 phosphorylation and decreased SQSTM1/p62 abundance, confirming the induction of autophagy (Fig. 8A). Torin 2 treatment also decreased GPX4 abundance (Fig. 8A), likely caused by the inhibition of protein synthesis. This finding motivated us to investigate the combined treatment of BC cells with Torin 2 and Fin56. To obtain IC_50_ values for combination analysis, we treated 253J and T24 cells with different concentrations of Torin 2 for 72 h. Torin 2 displayed high cytotoxicity (Fig. 8B). Next, we analyzed GPX4 expression upon single and combined treatments with Torin 2 and Fin56. We observed that the single treatments reduced GPX4 protein levels, with the most-prominent effect for the combined treatment (Fig. 8C). To investigate whether Torin 2 and Fin56 exhibit a synergistic effect on cytotoxicity, we performed isobologram analysis using the Chou-Talalay method (Fig. 8D) [49]. The combination of Torin 2 and Fin56 was synergistic at all tested concentrations in both BC cell lines (Fig. 8E and S5). Collectively, mTOR inhibition synergistically sensitizes BC cells to Fin56-induced ferroptosis.

In conclusion, our results show that Fin56 induces autophagy-dependent ferroptosis in BC cells and that the pharmacological or genetic inhibition of autophagy protects cells from ferroptosis induced by Fin56, whereas the inhibition of mTOR synergistically increases Fin56-induced ferroptosis.

## Discussion

BC is one of the most-common genitourinary cancers. Even with improvements in multimodal treatment strategies, such as (neo)adjuvant chemo-/immunotherapy, the therapeutic effects are far from satisfactory. In this study, we found that the type 3 ferroptosis inducer Fin56 triggers autophagy-dependent ferroptosis in BC cells. We also found that autophagy inhibition attenuates ferritin and GPX4 degradation, and alleviates oxidative damage induced by Fin56. In addition, we showed that the combination of Fin56 and Torin 2 results in synergistic cytotoxicity in BC cells.

Accumulating evidence indicates that ferroptosis represents a type of autophagy-dependent cell death [28, 50]. Several types of selective autophagy are involved in ferroptosis, such as clockophagy, ferritinophagy, lipophagy, and CMA [15, 29, 51, 52]. By evaluating the abundance of the autophagy markers LC3 and SQSTM1/p62 and the localization pattern of mRFP-EGFP-rLC3, we found that Fin56 induces autophagy in BC cells. Since LC3 and SQSTM1/p62 are common autophagy markers that participate in various types of autophagy, future studies will have to clarify whether Fin56 induces selective autophagy or just non-selective bulk autophagy.

Different types of ferroptosis inducers utilize different mechanisms to trigger this pathway. Fin56, a type 3 inducer, leads to ferroptosis mainly by promoting GPX4 degradation [14]. However, the mechanism(s) underlying this degradation are incompletely understood. Our study shows that Fin56 decreases GPX4 abundance, whereas autophagy inhibition at the nucleation stage (SAR405), at the expansion stage (ATG3 deficiency) or at the fusion stage (bafilomycin A_1_) attenuates this degradation process. Similarly, we demonstrated that Fin56 treatment leads to ferritin degradation in both MEFs and human BC cells, and that the degradation was damped by autophagy inhibition. We also found that bafilomycin A_1_ treatment alone reduces ferritin abundance (Fig. 5B). It has been reported that lysosomal vATPase inhibition causes functional iron deficiency by sequestering iron in the lysosomes and that iron deficiency causes ferritin degradation to maintain the balance of cellular iron [53]. As Fenton reaction is the main mechanism contributing to the generation of oxygen radicals, especially hydroxyl radicals, that in turn incite and propagate lipid peroxidation, careful control of iron levels is required to prevent Fenton reaction. GPX4 synthesis might decrease when cellular oxidative stress is relatively low. This could partially explain the reduction of GPX4 upon bafilomycin A_1_ treatment alone (Fig. 6B).

The specific mechanisms of Fin56-induced GPX4 degradation have not been elucidated so far. Shimada et al. [14] reported that Fin56-induced GPX4 degradation could be prevented by the inhibition of acetyl-CoA carboxylase (ACC). For yeast, it has been recently shown that ACC-dependent lipogenesis promotes autophagy [54]. Accordingly, it might be that autophagy represents the missing link between ACC activity and GPX4 degradation. A recent report indicates that erastin promotes CMA-mediated degradation of GPX4 by increasing the levels of lysosome-associated membrane protein 2a [29]. Generally, this report is consistent with our results using Fin56, and highlights the important role of autophagy for ferroptosis. However, we also found changes in LC3 and SQSTM1/p62 levels, and these proteins are not necessarily involved in CMA. We also observed that GPX4 degradation was abolished in *atg3* KO MEFs and that GPX4 co-localizes with LC3 upon Fin56 treatment, further suggesting that rather (macro)autophagy than CMA mediates Fin56-induced reduction of GPX4. Liu et al. [30] observed that RSL3 causes GPX4 degradation in human pancreatic cancer cells. Notably, hydroxychloroquine inhibited RSL3-induced GPX4 degradation only in PANC1 cells, but not in MIAPaCa2 cells [30]. Similarly, hydroxychloroquine could not prevent GPX4 degradation induced by mTOR inhibition [30]. These data indicate that GPX4 degradation does not rely on autophagy at least in the analyzed pancreatic cell lines. In our hands, Fin56-induced GPX4 degradation was inhibited by bafilomycin A_1_, clearly supporting an autophagy-dependency for Fin56 in our model system. With regard to mTOR signaling, similar differences become obvious. It has been suggested that (i) mTORC1 inhibition synergizes with ferroptosis inducers to suppress tumor growth [31], (ii) mTORC1 inhibition decreases GPX4 levels [30, 31] and (iii) RSL3 blocks mTOR activation [30]. Whereas we also detect synergistic effects between Fin56 and Torin 2 on the viability of BC cell lines and a Torin 2-mediated reduction of GPX4 levels, we do not observe an effect of Fin56 treatment on mTOR activation. It appears that Fin56 induces mTOR-independent autophagy, but that Fin56-induced ferroptosis can be supported by mTOR inhibition-mediated autophagy. Collectively, these data already indicate a rather complex and possibly stimulus-/cell-dependent relationship between autophagy and ferroptosis. We observed autophagy-dependency in two of four tested BC cell lines, and this heterogeneity has also been reported for erastin and RSL3 with regard to LC3-II accumulation, SQSTM1/p62 downregulation, and GPX4 degradation [27]. Fin56-induced ferroptosis was inhibitable in two cell lines. Of note, erastin-induced cell death was bafilomycin A_1_-sensitive in the identical two cell lines, indicating that the autophagy-dependency of ferroptosis signaling might be independent of the inducing stimulus. Generally, we observed different “categories” of Fin56-induced ferroptosis in our BC cells (Figure S6): (1) autophagy-dependent GPX4 degradation (253J, T24), (2) potentially autophagy-independent GPX4 degradation (J82; Figs. 1 and 7A), and (3) independent of GPX4 degradation (RT-112; Figs. 1 and 7A). It has already been proposed that Fin56-induced ferroptosis can be mediated—next to GPX4 degradation—via squalene synthase activation and coenzyme Q_10_ depletion [14], which might explain the observed phenotype in RT-112 cells. Future transcriptomic and proteomic analysis of our cell lines might reveal predictive factors for autophagy-dependent ferroptosis signaling.

Ferroptosis has attracted overwhelming interest in cancer research in recent years [55]. Since our results suggested that autophagy supports Fin56-induced ferroptosis, we validated the therapeutic benefit of the combination of Fin56 with autophagy activation, i.e., mTOR inhibition. Fin56 synergized with Torin 2 at all tested concentrations in BC cells. These findings constitute a promising proof-of-principle study to establish the combination of ferroptosis inducers with autophagy inhibitors for BC treatment.

## Materials and methods

### Reagents

Erastin (#S7242), RSL3 (#S8155), Fin56 (#S8254), SAR405 (#7682), Torin 2 (#S2817) were purchased from Selleck Chemicals (Houston, TX, USA); bafilomycin A_1_ (#B1793), α-tocopherol (#258024), and Liproxstatin-1 (#SML-1414) were purchased from Sigma-Aldrich (St. Louis, MO, USA). In addition, the following reagents were used: dimethyl sulfoxide (DMSO; AppliChem GmbH, #A3672), milk powder (Carl Roth, #T145.2), phosphate-buffered saline (PBS) (Thermo Fisher Scientific/Gibco, #14190-094), 0.05% trypsin/EDTA solution (Thermo Fisher Scientific/Gibco, #25300-062), puromycin (InvivoGen, #ant-pr-1).

For immunoblotting, primary antibodies against SQSTM1/p62 (PROGEN Biotechnik, #GP62-C), LC3 (Cell Signaling Technology, #2775), ACTB/β-actin (Sigma-Aldrich, #A5316), phospho-mTOR Ser2448 (Cell Signaling Technology, #2971), mTOR (Cell Signaling Technology, #2972), phospho-ULK1 Ser757 (Cell Signaling Technology, #6888), ULK1 (clone D8H5, Cell Signaling Technology, #8054), tubulin (Sigma, #T5168), ATG3 (Cell Signaling Technology, #3415), SLC7A11 (previously described in ref. [56]), ferritin (Abcam, #ab75973), HA Tag (clone 3F10, Roche Applied Science, #11867423001), GPX4 (Abcam, #ab125066), GAPDH (Abcam, #ab8245) were used. IRDye 680- or IRDye 800-conjugated secondary antibodies (#926-68077, #926–68070/71, #926-32210/11) were purchased from LI-COR Biosciences.

For immunofluorescence, primary antibodies against TOM20 (Santa Cruz Biotechnology, #17764) and LC3B (MBL, #PM036) were used. Alexa Fluor^®^ 488-conjugated and Alexa Fluor^®^ 647-conjugated secondary antibodies were purchased from Jackson ImmunoResearch Laboratories (#115-545-003 and #111-605-003). DAPI was obtained from Roth (#6335.1).

### Cell lines and cell culture

All BC cell lines used in this research (kindly provided by Margaretha Skowron, Department of Urology, Heinrich Heine University Düsseldorf, Düsseldorf, Germany) were cultured in Dulbecco’s Modified Eagle Medium (DMEM) (Thermo Fisher Scientific/Gibco, #41965) supplemented with 10% fetal calf serum (FCS; Sigma-Aldrich, #F0804) and 4.5 g/l d-glucose at 37 °C and 5% CO_2_ humidified atmosphere. *ulk1/ulk2* DKO MEFs (kindly provided by Tullia Lindsten, Memorial Sloan Kettering Cancer Center, New York City, USA), MYC-ULK1-expressing *ulk1/ulk2* DKO MEFs (generation see below), *atg3* KO and *Atg3* WT MEFs (kindly provided by Masaaki Komatsu, Department of Organ and Cell Physiology, Juntendo University School of Medicine, Tokyo, Japan), and Flag-Strep-HA-GPX4 MEFs (previously described in ref. [47]) were cultured in DMEM supplemented with 10% FCS, 100 U/ml penicillin and 100 µg/ml streptomycin (Thermo Fisher Scientific/Gibco, 15140) at 37 °C and 5% CO_2_ humidified atmosphere.

### Cell viability assay

Cell viability was measured using MTT assay. In all, 5000 cells per well were seeded on a 96-well plate one day prior to the experiment. The following day, the cells were treated with different compounds as described above. After that, 0.5 mg/ml MTT (Roth, #4022) was added to the cells, and cells were incubated at 37 °C for 1 h. Then the plates were centrifuged at 600 rcf for 5 min, and formazan crystals were dissolved in DMSO for 20 min in dark. The absorbance was measured at test (570 nm) and reference (650 nm) wavelengths using a microplate reader (BioTek, Synergy Mx). The mean of the absorbance of untreated control samples was set as 100%.

### siRNA transfection

For *Ulk1* knockdown, wild-type MEFs (at 50–60% confluence) in opti-MEM medium (Thermo Fisher Scientific, #31985062) were transfected with DharmaFECT 1 reagent (Dharmacon, #T-2001-03) using 25 nM of ON-TARGETplus Mouse ULK1 siRNA–SMARTpool (Dharmacon, #L-040155-00-0010) or ON-TARGETplus Non-targeting Control Pool (Dharmacon, #D-001810-10-20). For *GPX4* knockdown, BCs at 70–80% confluence were transfected with DharmaFECT 1 reagent using 25 nM of ON-TARGETplus Human GPX4 siRNA–SMARTpool (Dharmacon, #L-011676-00-0005) or non-targeting control pool. After 24 h, cells were seeded for cell viability assay and immunoblotting.

### Retroviral transduction

cDNA encoding human SLC7A11 was amplified from 253J cDNA by PCR using the following primers: fwd: CCCCCGTGTGTCCCTACTA, rev: GGCAGATTGCCAAGATCTCAA. Subsequently, the SLC7A11 cDNA harboring 5′ and 3′ overlap to pMSCVpuro sequences was amplified and directly cloned into pMSCVpuro (Clontech Laboratories, Takara Bio, #631461) by sequence and ligation-independent cloning (SLIC; [57]). The following primers were used: HA-SLC7A11 fwd: GCCGCCACCATGTACCCATACGATGTTCCAGATTACGCTGTCAGAAAGCCTGTTGTG, HA-SLC7A11 rev: TACCCGGTAGAATTCTCATAACTTATCTTCTTCTGG, pMSCV fwd: GAAGATAAGTTATGAGAATTCTACCGGGTAGG, and pMSCV rev: AGCGTAATCTGGAACATCGTATGGGTACATGGTGGCGGCGAATTCGTTAACCTCGAG. Human GPX4 and 3’-UTR of GPX4 were amplified from Jurkat cell cDNA using the following primers: fwd: GCTGGACGAGGGGAGGAG, rev: CACAAGGTAGCCAGGGGTG. They were subsequently cloned into pMSCVpuro. MYC-mULK1 cDNA was a gift from Do-Hyung Kim (Addgene plasmid #31960; http://n2t.net/addgene:31960; RRID:Addgene_31960). To generate the pMSCVblast/MYC-mULK1 vector, *MYC-mULK1* was amplified with the primers 5′ gattaactcgagATGGAGCAAAAGCTCATTTCTGAGG 3′ and 5′ ctagttgttaacTCAGGCATAGACACCACTCAGC 3′, digested with Xho1 and Hpa1 (Thermo Scientific, #FD0694 and #FD1034), and cloned into pMSCVblast digested with the same enzymes. Plat-E cells (kindly provided by Toshio Kitamura, Institute of Medical Science, University of Tokyo, Japan) were used as packaging cells and were transfected with the retroviral expression vectors pMSCVpuro/HA-SLC7A11, pMSCVpuro/mRFP-EGFP-rLC3 (previously described in ref. [58]), pMSCVpuro/hGPX4 or pMSCVblast/MYC-mULK1 using FuGENE^®^ 6 (Roche, #11988387001). For the retroviral infection of human cell lines, pVSVG was co-transfected into Plat-E cells. After 48 h, human BC cells or *ulk1/ulk2* DKO MEFs were incubated with the corresponding retroviral supernatants containing 3 µg/ml Polybrene (Sigma-Aldrich, #H9268-106) and selected in a medium containing either 2.5 µg/ml puromycin (InvivoGen, #ant-pr-1) or 35 µg/ml blasticidin (InvivoGen, #ant-bl-05), respectively.

### Immunoblotting

Cells were harvested by scraping and lysed in ice-cold lysis buffer (20 mM Tris/HCl, pH 7.5, 150 mM NaCl, 0.5 mM EDTA, 1% [v/v] Triton X-100, 1 mM Na_3_VO_4_, 10 μM Na_2_MoO_4_, 2.5 mM Na_4_P_2_O_7_, 10 mM NaF and protease inhibitor cocktail [Sigma-Aldrich, #P2714]). Equal amounts of proteins were subjected to 8–15% sodium dodecyl sulphate–polyacrylamide gel electrophoresis. Proteins were then transferred to polyvinylidene fluoride membranes (Millipore, #IPFL00010) and analyzed using the indicated primary antibodies and appropriate IRDye-conjugated secondary antibodies. Protein signals were detected using an Odyssey Infrared Imaging system (LI-COR Biosciences) and quantified using Image Studio lite 4.0 (LI-COR Biosciences).

### Immunofluorescence

In all, 5 × 10^4^ cells were plated on glass coverslips one day prior to treatment. After treatment, the cells were fixed on ice using 4% paraformaldehyde-FBS for 15 min, quenched with 50 mM NH_4_Cl for 15 min, and permeabilized with 50 µg/ml digitonin (Roth, #4005) for 5 min. Subsequently, the cells stably expressing mRFP-EGFP-rLC3 were directly stained with DAPI. The other cells were blocked with 3% bovine serum albumin (Roth, #8076)-PBS for 30 min, and then incubated with indicated primary antibodies and corresponding secondary antibodies for 1 h each. Cells were embedded in ProLong Glass Antifade Mountant (Thermo Fisher Scientific, #P36980) containing DAPI. For BODIPY 581/591 C11 staining, 2.4 × 10^4^ of 253J or T24 cells per well were seeded on ibidi µ-Slide 8 Well (ibidi #80826). The following day, cells were treated with Fin56 (2 µM) or DMSO for 4 h. After treatment, cells were incubated with 2 μM BODIPY 581/591 C11 (Thermo Fisher Scientific, #D3861) for 30 min. Subsequently, cells were washed twice with PBS and subjected to microscopy. The Zeiss Axio Observer 7 fluorescence microscope (Zeiss, Köln, Germany) with a Plan Apochromat ×40/1.4 oil-objective (Zeiss, Köln, Germany) was used for imaging. The images were quantified with ImageJ, and the macros used for quantification are listed in the supplementary methods.

### DCF assay

In all, 5000 cells per well were seeded on a 96-well plate one day prior to the experiment. The following day, the cells were treated with different compounds as described above. After that, cells were washed once with PBS and treated with 10 μM H_2_DCFDA (Thermo Fisher Scientific, #D399) at 37 °C for 30 min in dark. Then, the cells were washed twice with PBS, and the DCF fluorescence was measured immediately using excitation (490 nm) and emission (520 nm) wavelengths using a microplate reader (BioTek, Synergy Mx).

### Assessment of lipid peroxidation using BODIPY 581/591 C11 by flow cytometry

In all, 2 × 10^6^ cells per well were seeded on 6-well dishes one day prior to the experiment. On the next day, cells were treated with the indicated concentration of Fin56 to induce ferroptosis. Cells were incubated with 1.5 µM BODIPY 581/591 C11 for 30 min at 37 °C before they were harvested by trypsinisation. Subsequently, cells were resuspended in 500 μl of fresh PBS and analyzed using an LSRFortessa flow cytometer (BD, Franklin Lakes, NJ, USA). The 488-nm laser was used for excitation. Data was collected using the FL1 detector with a 530/30 BP filter. A total of 20,000 events were acquired for each sample.

### Statistics

For immunoblotting, the density of each protein band was divided by the average density of all bands of this protein. The ratios were normalized to the loading control, and fold changes were calculated by dividing each normalized ratio by the control line (fold change is 1 for the control lane, *n* ≥ 2). The results are shown as mean ± standard deviation in the bar diagrams and in the dose–response curves, and *P* values were determined by ordinary one-way analysis of variance (ANOVA) with Dunnett’s post hoc test. For cell viability assay, results are shown as mean ± standard deviation in the bar diagrams, and *P* values were determined by two-way ANOVA with Sidak’s post hoc test. All IC_50_ values were calculated using GraphPad Prism 7.01. Compusyn 1.0 (ref. [59]) was used to perform isobologram analysis for the combination of two compounds. The resulting combination index (CI) values represent the combined effects of two compounds and are interpreted as synergistic (CI < 1), additive (CI = 1), or antagonistic (CI > 1) [59]. For the quantification of puncta in mRFP-EGFP-rLC3-expressing cells, the images were quantified with ImageJ. The numbers of puncta in Fin56 treatment are normalized to the numbers of puncta in medium treatment. The results are shown as mean + standard deviation in the bar diagrams, and *P* values were determined by two-way ANOVA with Sidak’s post hoc test.

## Supplementary information

supplementary material

## Data Availability

The data that support the findings of this study are available from the corresponding author, B.S., upon reasonable request.
