# Supplementary material for: Fin56-induced ferroptosis is supported by autophagy-mediated GPX4 degradation and functions synergistically with mTOR inhibition to kill bladder cancer cells
Source: Cell Death Dis. 2021 Oct 29;12(11):1028. doi: 10.1038/s41419-021-04306-2 (PMC8556316; doi:10.1038/s41419-021-04306-2)
Supplement: Supplementary file 1 — supplementary material [file 41419_2021_4306_MOESM1_ESM.pdf]

Fin56-induced ferroptosis is supported by autophagy-mediated GPX4 degradation and functions synergistically with mTOR inhibition to kill bladder cancer cells

Yadong Sun[1], Niklas Berleth[1], Wenxian Wu[1], David Schlütermann[1], Jana Deitersen[1], Fabian Stuhldreier[1], Lena Berning[1], Annabelle Friedrich[1], Seda Akgün[1], María José Mendiburo[1], Sebastian Wesselborg[1], Marcus Conrad[2,3], Carsten Berndt[4], Björn Stork[1,*]

[1]Institute of Molecular Medicine I, Medical Faculty, Heinrich Heine University, Düsseldorf, Germany

[2]Institute of Metabolism and Cell Death, Helmholtz Zentrum München, Neuherberg, Germany

[3]Pirogov Russian National Research Medical University, Laboratory of Experimental Oncology, Ostrovityanova 1, Moscow 117997, Russia

[4]Department of Neurology, Medical Faculty, Heinrich Heine University, Düsseldorf, Germany

Suppl. Figures S1-S6

Suppl. Methods

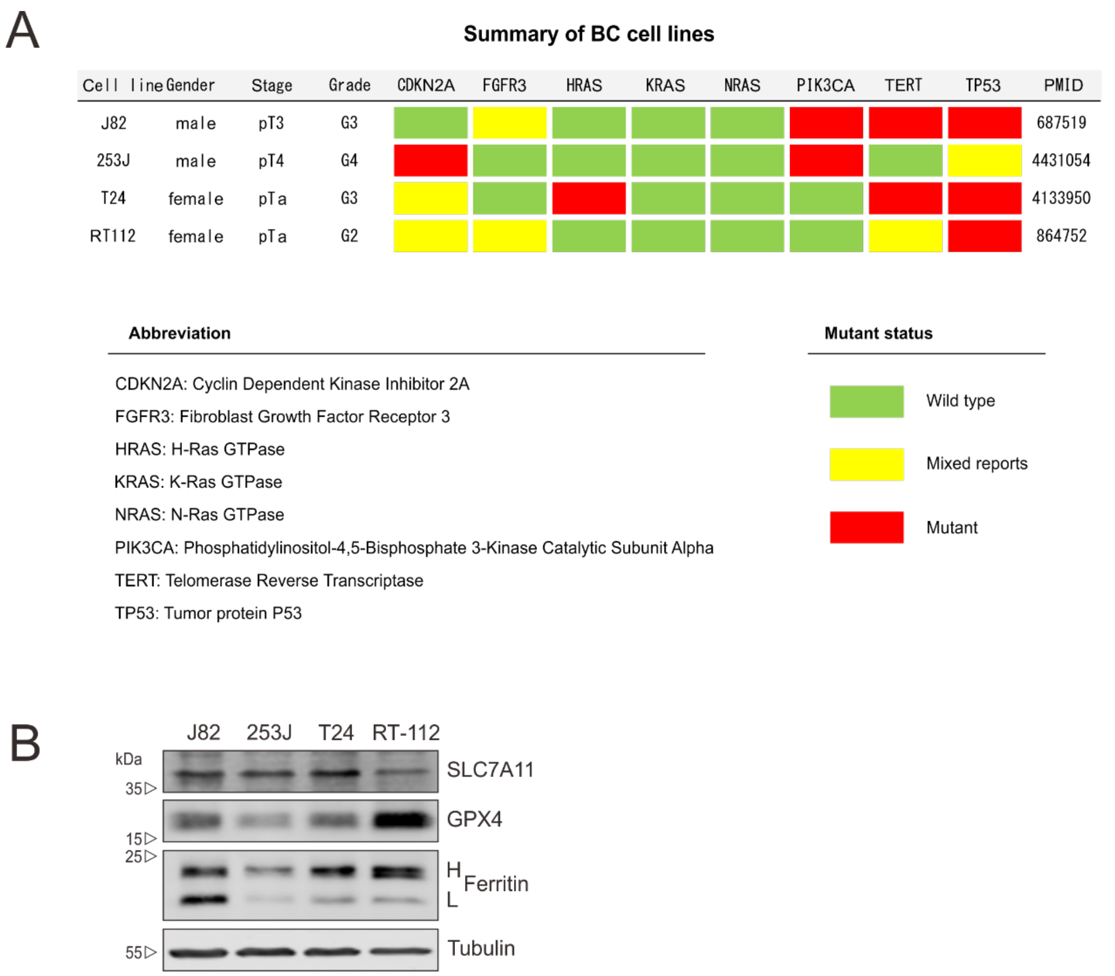

Figure S1 (related to main figure 1): Characterization of bladder cancer cell lines. (**A**) Summary of bladder cancer cell lines used in this study. Cell line information is an excerpt from table 2 in “Systematic Review: Characteristics and Preclinical Uses of Bladder Cancer Cell Lines” by Zuiverloon et al. (doi 10.333/BLC-180167), used under CC BY-NC 4.0. (**B**) Cleared cellular lysates of J82, 253J, T24 and RT-112 cells were prepared and subjected to immunoblotting for SLC7A11, GPX4, Ferritin (H, heavy chain; L, light chain), and tubulin.

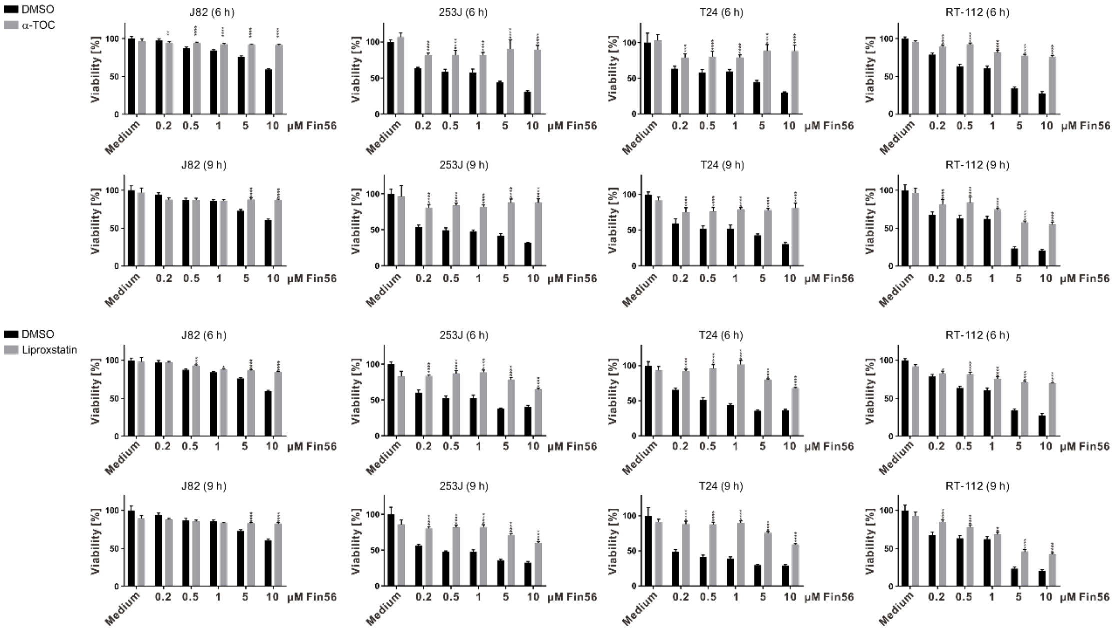

Figure S2 (related to main figure 2B): Fin56 induces ferroptosis in bladder cancer cells. J82, 253J, T24 and RT-112 cells were treated with indicated concentrations of Fin56 with or without α-Tocopherol (α-TOC, 100 µM) or Liproxstatin-1 (500 nM) for 6 or 9 h. After treatment, cell viability was measured using MTT assay. Results are shown as means ± SD of two independent experiments performed in triplicates for each treatment. P values were determined by two-way ANOVA with Sidak´s post hoc test. ****$p < 0.0001$.

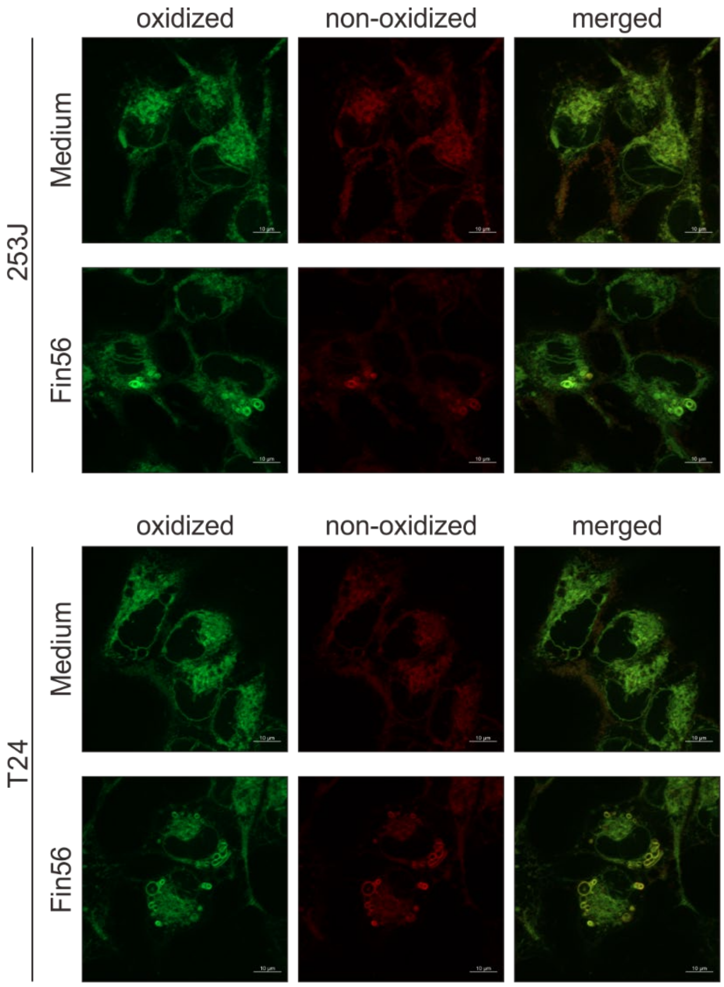

Figure S3 (related to main figure 5B): Fin56 induces “onion-like” vesicular structures in 253J and T24 cells. 253J and T24 cells were treated with Fin56 (2 μM) or DMSO for 4 h. After treatment, cells were incubated with 2 μM BODIPY (581/591) C11 for 30 min. Subsequently, cells were washed twice with PBS. Staining was detected using Zeiss Axio Observer 7 fluorescence microscope with a Plan Apochromat 40x/1.4 oil objective. 475 nm or 555 nm excitation filters were used to detect oxidized or non-oxidized forms of BODIPY (581/591) C11-stained structures.

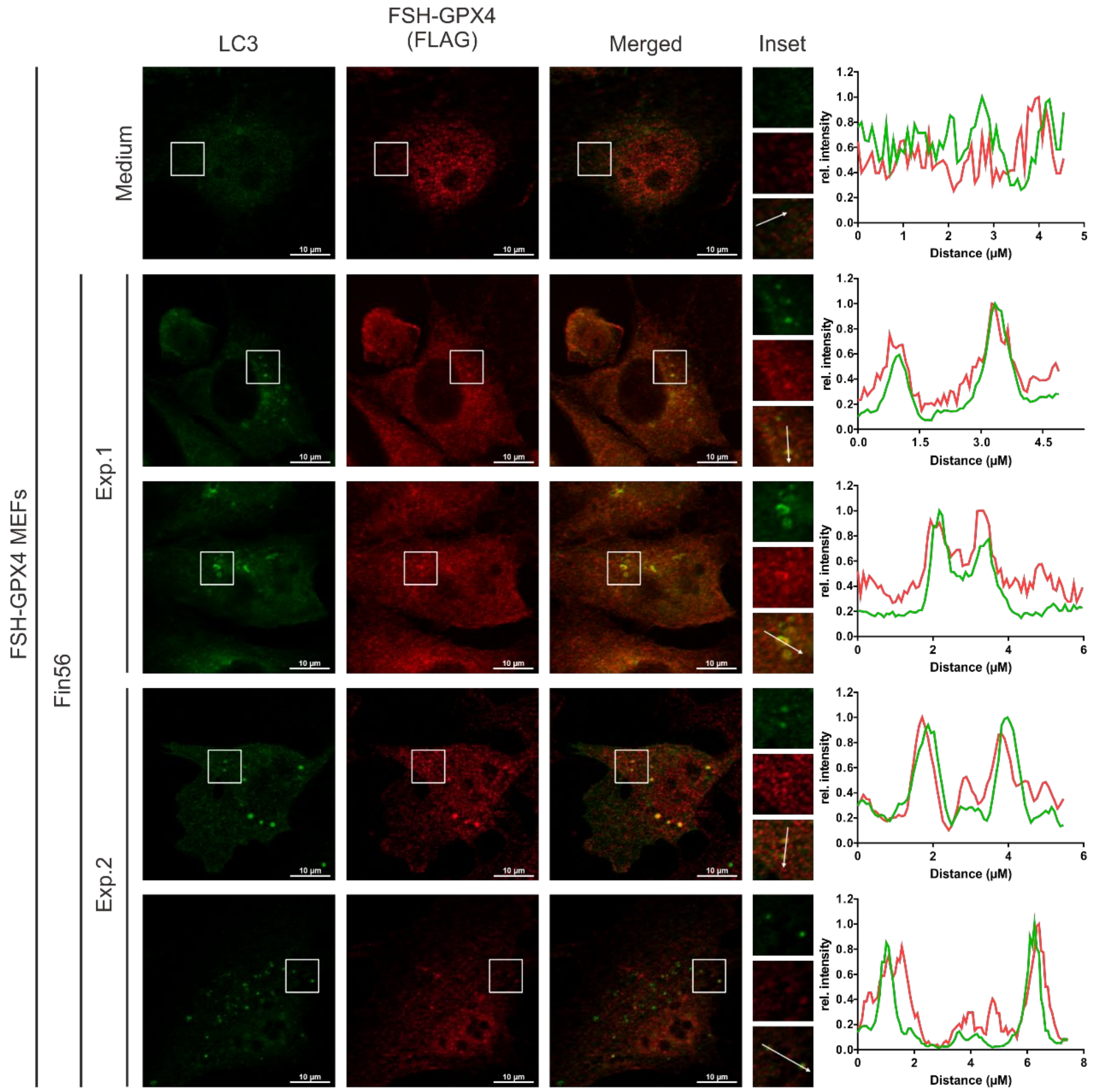

Figure S4 (related to main figure 6): GPX4 co-localizes with LC3 upon Fin56 treatment. FSH-GPX4-expressing MEFs were grown on glass cover slips one day prior to treatment. The following day, cells were treated with 5 µM Fin56 for 6 h. After treatment, cells were fixed, permeabilized and incubated with indicated antibodies (LC3: MBL, #PM036; FLAG: Sigma, #F1804). Representative sections of two independent experiments are depicted. The bar graphs represent the pixel intensities of the areas indicated by the respective white arrows shown in the insets.

A

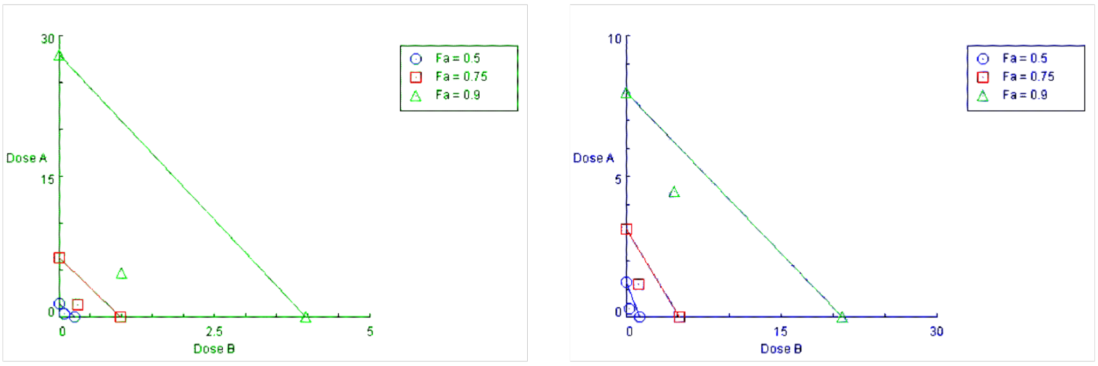

253J

T24

B

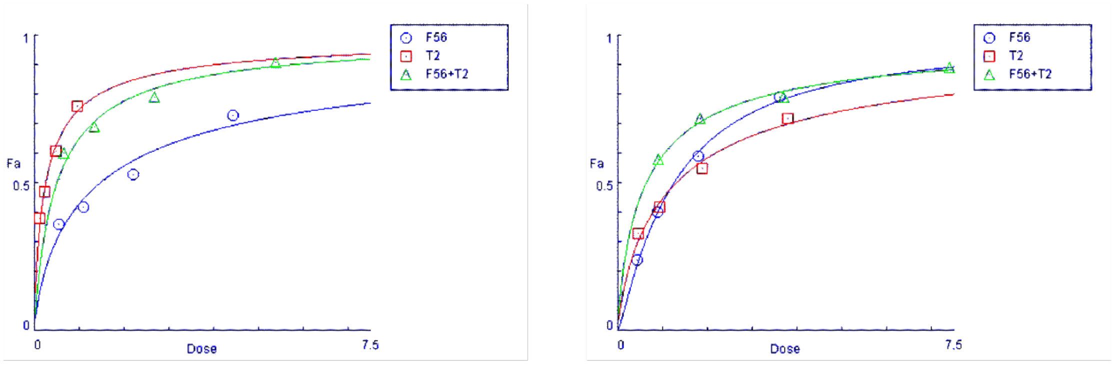

253J

T24

Figure S5 (related to main figure 8E): mTOR inhibition synergistically sensitizes BCs to Fin56-induced ferroptosis. Depicted are isobolograms of Torin 2 in combination with Fin56.

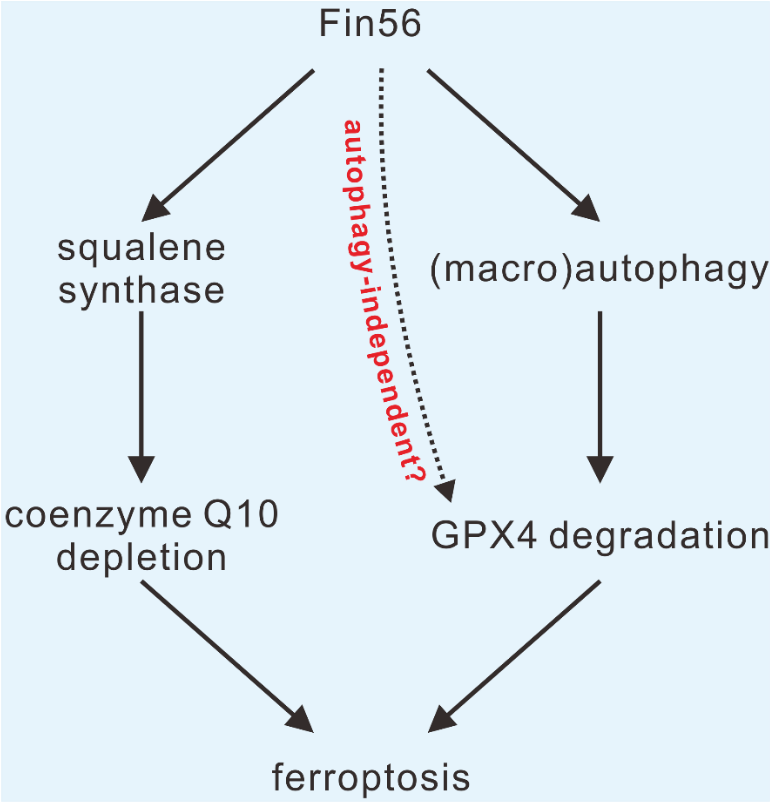

Figure S6: Fin56-induced ferroptosis. In this study, we showed that Fin56 can induce GPX4 degradation via (macro)autophagy. Next to this pathway, Fin56 can induce ferroptosis independently of GPX4 degradation (ref. 16 in main manuscript). Finally, we cannot exclude that GPX4 degradation might also occur in an autophagy-independent manner. GPX4, Glutathione peroxidase 4. Scheme modified from Shimada et al. (ref. 16 in main manuscript).

Supplementary Methods

Macro for quantification of mRFP-EGFP-rLC3 puncta and DAPI positive nuclei

- dir1 directory to original images, dir2 directory to processed images

```
dir1 = getDirectory("Choose a Directory ");
dir2 = getDirectory("Choose a Directory ");
setBatchMode(true);
list = getFileList(dir1);
for (i=0; i<list.length; i++)
	{
		open(dir1+list[i]);
		title = File.nameWithoutExtension ;
						//split and save channels
						run("Options...", "iterations=1 count=1 black do=Nothing");
						run("Split Channels");
						saveAs("Tiff", dir2+title+"_DAPI.tif");
						close();
						run("8-bit");
						saveAs("Tiff", dir2+title+"_GFP-LC3.tif");
						close();
						saveAs("Tiff", dir2+title+"_RFP-LC3.tif");
						close();
				//process and count nuclei
				open(dir2+title+"_DAPI.tif");
				run("Median...", "radius=20");
				run("Subtract Background...", "rolling=150");
				run("Enhance Contrast...", "saturated=0.01 normalize");
				run("8-bit");
				run("Auto Threshold", "method=Li white");
				run("Fill Holes");
				run("Adjustable Watershed", "tolerance=1.5");
				saveAs("Tiff", dir2+title+"_DAPI_processed.tif");
				run("Analyze Particles...", "size=30-Infinity pixel show=Outlines exclude clear
summarize");
				saveAs("Tiff", dir2+title+"_DAPI_drawing.tif");
				close();
				open(dir2+title+"_DAPI.tif");
				run("8-bit");
				saveAs("Tiff", dir2+title+"_DAPI.tif");
				close();
					//process and count RFP+ dots
					open(dir2+title+"_RFP-LC3.tif");
```

```
                    run("Convoluted Background Subtraction", "convolution=Median
radius=15");
                    run("8-bit");
                    setThreshold(45, 255);
                    saveAs("Tiff", dir2+title+"_RFP-LC3_processed.tif");
                    run("Analyze Particles...", "size=0.01-Infinity pixel show=Outlines
exclude clear summarize add");
                    saveAs("Tiff", dir2+title+"_RFP-LC3_drawing.tif");
                    close();
                    //save ROIs
                    roiManager("save", dir2+title+"Manager.zip")
                    //process and count GFP+ dots
                    open(dir2+title+"_GFP-LC3.tif");
                    run("Convoluted Background Subtraction", "convolution=Median
radius=15");
                    run("8-bit");
                    setAutoThreshold("Default dark");
                    run("Threshold...");
                    setThreshold(35, 255);
                    setOption("BlackBackground", false);
                    run("Convert to Mask");
                    run("Close");
                    //select all ROIs in ROI manager
                            count=roiManager("count");
                            array=newArray(count);
                            for(f=0; f<count;f++)
                            array[f] = f;
                            roiManager("Select", array);
                    //roiManager("Show None");
                    roiManager("Show All");
                    roiManager("Measure");
                    roiManager("delete")
                    saveAs("Tiff", dir2+title+"_GFP-LC3_drawing.tif");
                    close();
            //name result sheet
                name = getTitle;
                dotIndex = indexOf(name,".");
                name = substring(name, 0, dotIndex);
            close();
            //save result sheet
            selectWindow("Results");
            saveAs("Results", dir2+name+".txt");
}
setBatchMode(false);
```

```
//save summary sheet
selectWindow("Summary");
saveAs("Text", dir2+"Summary.txt");
run("Close All")
showMessage("Well done!!");
exit();
```
